# Deep learning and citizen science enable automated plant trait predictions from photographs

**DOI:** 10.1038/s41598-021-95616-0

**Published:** 2021-08-12

**Authors:** Christopher Schiller, Sebastian Schmidtlein, Coline Boonman, Alvaro Moreno-Martínez, Teja Kattenborn

**Affiliations:** 1 Institute of Geography and Geoecology, Karlsruhe Institute of Technology (KIT), 76131 Karlsruhe, Germany; 2 Department of Environmental Science, Institute for Water and Wetland Research, Radboud University, Nijmegen, The Netherlands; 3 Image Processing Laboratory (IPL), Universitat de València, Valencia, Spain; 4 Remote Sensing Center for Earth System Research, Leipzig University & Helmholtz Centre for Environmental Research (UFZ), Leipzig, Germany

**Keywords:** Ecosystem ecology, Biogeography, Macroecology, Environmental sciences, Environmental impact

## Abstract

Plant functional traits (‘traits’) are essential for assessing biodiversity and ecosystem processes, but cumbersome to measure. To facilitate trait measurements, we test if traits can be predicted through visible morphological features by coupling heterogeneous photographs from citizen science (iNaturalist) with trait observations (TRY database) through Convolutional Neural Networks (CNN). Our results show that image features suffice to predict several traits representing the main axes of plant functioning. The accuracy is enhanced when using CNN ensembles and incorporating prior knowledge on trait plasticity and climate. Our results suggest that these models generalise across growth forms, taxa and biomes around the globe. We highlight the applicability of this approach by producing global trait maps that reflect known macroecological patterns. These findings demonstrate the potential of Big Data derived from professional and citizen science in concert with CNN as powerful tools for an efficient and automated assessment of Earth’s plant functional diversity.

## Introduction

Global change driven by global warming, land-cover conversion and landscape fragmentation imposes a threat on global biodiversity^1^. The loss of biodiversity inevitably leads to a loss of ecosystem functioning and processes^1^, which are essential to human well-being^2^. Ecosystem functioning, in turn, can be assessed using plant functional traits (hereafter: ’traits’), since it results from the trait composition of the species that compose a plant community^3^. Therefore, the impacts of environmental changes on ecosystem functioning can most simply be assessed on species level using traits^4–7^. These traits can, for instance, be related to plant size, e.g. leaf area or growth height, or tissue constituents, e.g. leaf nitrogen concentration or stem specific density. Such traits, though, have in common a high measurement effort. Thus, an effective trait measurement tool would greatly facilitate rapid ecosystem monitoring^1^, which is urgently needed in light of expected trait shifts around the globe^8^.

Convolutional Neural Networks (CNN), a deep learning-oriented computer vision technique, are evolving as promising tools for ecological research^9,10^, e.g. in plant community identification on unmanned aerial vehicle (UAV) images^11^ or species identification by harnessing plant photograph databases^12^. Particularly, the fundamental innovation of CNN is their efficiency in target-oriented learning of image features from raw input data. Hence, CNN may also enable to infer trait expressions from plant photographs by means of directly related morphological plant features or covariance with indirect causal links^13^ among visible and non-visible plant features. For instance, features such as the shape and thickness of plant leaves might be indicative of traits such as leaf nitrogen concentration and therefore be predictable by CNN.

Currently, this approach is hampered by the lack of a dataset comprising plant photographs with matching trait measurements that cover the global plant functional spectrum. In recent years, however, global data initiatives and citizen science projects have emerged as a strongly growing data source, which builds upon the collective effort of the scientific community^14,15^. Consequently, we employ a weakly supervised learning approach that combines the independent data sources of (1) the iNaturalist database providing a worldwide record of millions of plant photographs including taxa and geolocations^16^ and (2) the TRY database containing more than 11 million trait measurements across more than 270,000 taxa^7^.

**Figure 1:**
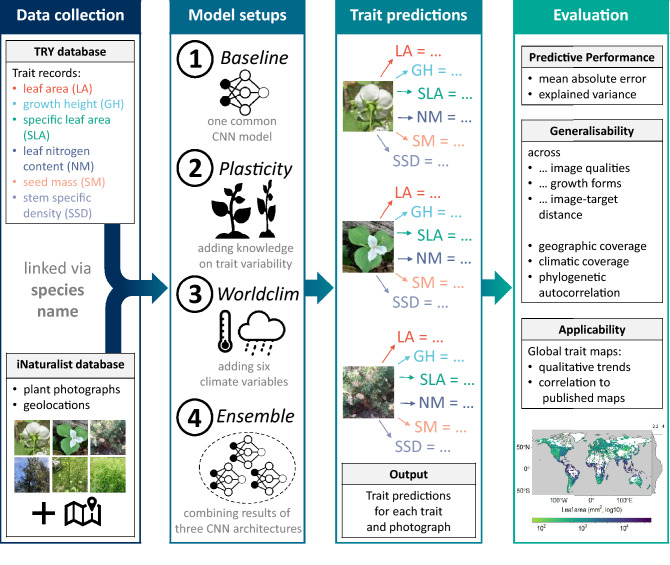
Conceptual diagram of the analyses, from data collection to evaluation. Data collection included linking plant functional trait records from the TRY database and plant photographs from the iNaturalist database via species names. The Convolutional Neural Networks were trained in four setups, which enabled trait predictions. The evaluation included analyses of predictive performance, generalisability and applicability. CNN, Convolutional Neural Networks.

With this setup, we test if it is possible to infer plant traits from simple RGB photographs using CNN (setup 1, Fig. 1). As intra-specific trait variability can be substantial along environmental gradients^17–19^, we used species-specific trait distributions rather than mean trait values in the CNN training in a second step (setup 2). Moreover, contextual cues might foster CNN predictive performance^10^. Consequently, bioclimatic variables were incorporated into the CNN due to their link to trait expressions (setup 3)^8,20,21^. Fourthly, we assessed the effect on predictive performance of an ensemble approach combining different CNN models (setup 4)^22^.

In addition to a statistical evaluation on independent observations, we assessed the plausibility of the trained CNN by global trait maps derived by applying each trait model on approx. 185,000 iNaturalist observations that were not included in the training process. This highlighted both a potential future application in light of an exponential growth of the iNaturalist database providing new plant photographs day by day, and the practical value of the presented approach. Therefore, we consider this study an important pioneering work to pave the way for rapid and efficient trait assessments.

## Results

We built datasets with image-trait couples for leaf area (LA), growth height (GH), specific leaf area (SLA), leaf nitrogen concentration (LNC), seed mass (SM) and stem specific density (SSD; for details see Supplementary Table 1). Some of these traits, such as LA and GH, could be readily visible by the computer vision models, whereas the others might be indirectly visible on account of the strong covariances between the traits investigated in this study^13^. Moreover, traits such as SLA and LNC might be predictable in photographs on account of the thickness, color intensity or shape of the plant’s leaves. We restricted the number of images per species to prevent the model from learning species-specific trait expressions. Simultaneously, we maximized the number of species in the dataset to achieve a wide range of trait expressions and a sufficiently large dataset. We applied a stratified sampling design (each species being a stratum) allowing up to eight images per species to acquire a sufficient amount of training data while balancing the taxonomic evenness (Table 1).

**Table 1:** Summary of plant functional trait datasets. Information on the maximum number of images per species, total number of species ($\mathrm {N_{species}}$), number of images with woody ($\mathrm {N_{woody}}$) and non-woody species ($\mathrm {N_{non-woody}}$), number of observations with non-zero standard deviations for Plasticity setup ($\mathrm {N_{TA}}$), number of images in training ($\mathrm {N_{training}}$), validation ($\mathrm {N_{validation}}$) and test ($\mathrm {N_{test}}$) dataset as well as total number of images ($\mathrm {N_{total}}$) in each trait dataset. LA, leaf area; GH, growth height; SLA, specific leaf area; LNC, leaf nitrogen concentration; SM, seed mass; SSD, stem specific density.

Trait	Max. images per species	$\mathrm {N}_{\mathrm {species}}$	$\mathrm {N}_{\mathrm {woody}}$	$\mathrm {N}_{\mathrm {non-woody}}$	$\mathrm {N}_{\mathrm {TA}}$	$\mathrm {N}_{\mathrm {training}}$	$\mathrm {N}_{\mathrm {validation}}$	$\mathrm {N_{test}}$	$\mathrm {N}_{\mathrm {total}}$
LA	8	1361	3937	6096	7773	7216	1804	1013	10,033
GH	2	8161	6862	8897	11,040	11,348	2836	1575	15,759
SLA	3	4615	7099	6041	9351	9461	2365	1314	13,140
LNC	3	4339	7103	5261	8254	8903	2225	1236	12,364
SM	1	9725	3903	5822	5404	7003	1750	972	9725
SSD	5	2455	10078	685	7176	7750	1937	1076	10,763

### Implementation of trait plasticity, bioclimatic data and ensembles

**Figure 2:**
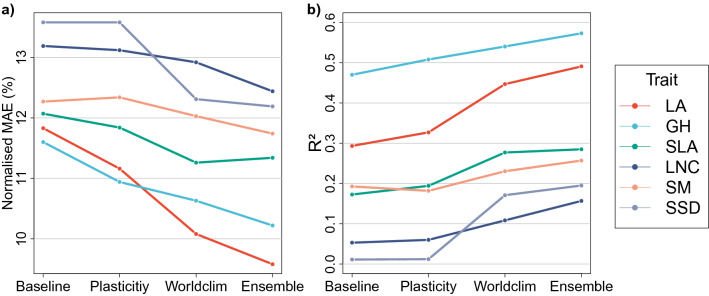
Results of the four model setups. Normalised mean absolute error (**a**) and $R^2$ (**b**) for the basic CNN model setup (’Baseline’), the setup including trait variability (’Plasticity’), the setup including bioclimatic data (’Worldclim’) and the ensemble setup (’Ensemble’). Mean absolute error was normalised by the respective test dataset’s range to enable a comparison between the predictive performance of the six traits. $R^2$ shows the explained variance of the linear fit of test predictions versus test targets. ^23^. LA, leaf area; GH, growth height; SLA, specific leaf area; LNC, leaf nitrogen concentration; SM, seed mass; SSD, stem specific density.

The prediction of the basic CNN (’Baseline’ setup) yielded normalised mean absolute errors (NMAE) between 13.6% (SSD) and 11.6% (GH; Fig. 2, Supplementary Table 2). The explained variance of the linear fit did not exceed 5% for SSD and LNC, but reached up to 47% for GH. Accounting for the intra-specific variability of traits by providing a distribution of trait values instead of a single mean value for each species (‘Plasticity’ setup) generally improved predictive performance with the exception of SM, increasing the explained variance by up to 3.79%-points (GH). Adding knowledge on the local climate of a photograph’s location (’Worldclim’ setup) generally increased the predictive performance. Its effect on $R^2$ was higher on LA, SLA and SSD (between approx. $+8$ and $+16$%-points) than on GH, LNC and SM (between approx. $+3$ and $+5$%-points). Furthermore, the ’Ensemble’ setup, in which we averaged the predictions of three common CNN architectures, generally increased $R^2$, yielding a rise of more than 4%-points in explained variance for LA and LNC.

**Figure 3:**
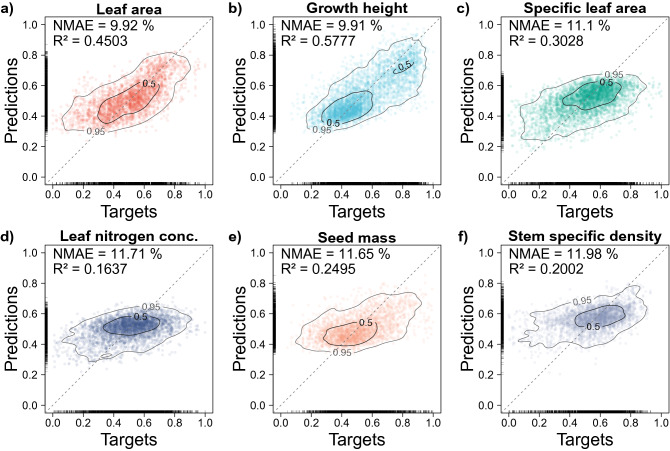
Distributions of predictions and targets of 3-fold cross-validation. **a-f**, All target-reference pairs (N = 3 × $\mathrm {N_{test}}$) of Ensemble setup of 3-fold cross-validation for the six plant functional traits leaf area (**a**), growth height (**b**), specific leaf area (**c**), leaf nitrogen concentration (**d**), seed mass (**e**) and stem specific density (**f**). Dashed grey lines indicate the one-to-one line for reference. Contour lines indicate the bivariate occurrence probabilities (50% and 95%) computed by kernel density estimation using R^23^ package ’ks’ (version 1.11.7)^24^.

The results of a threefold cross-validation based on the ensemble models revealed that the traits characterizing leaf form or habitus - GH and LA - demonstrated the lowest NMAE (9.91% and 9.92%; for prediction errors in original unit see Supplementary Table 3) and highest $R^2$ (.58 and .45; Fig. 3), whereas the traits related to tissue constituents, LNC and SSD, ranked lowest (11.71% and 11.98%; .16 and .2).

**Figure 4:**
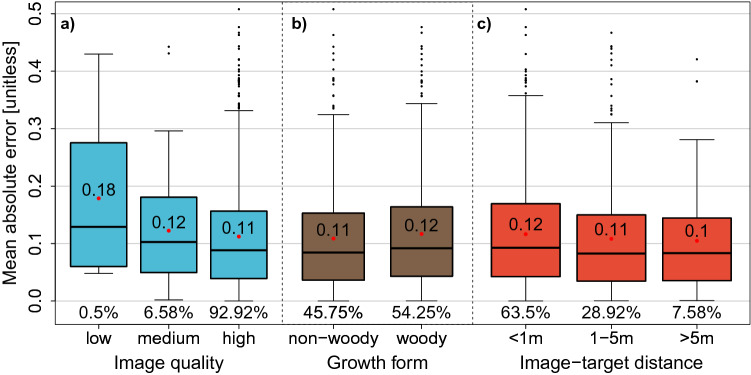
Model evaluation results. (**a-c**) Mean absolute errors across different image qualities (**a**), growth forms (**b**) and image-target distances (**c**) concerning 200 images per plant functional trait ($\hbox {N} = 1200$ images). None of the images of the evaluation dataset were used in the training phase. The percentage of images falling in a category is indicated below each box plot. Red dots and the number linked to them are the mean absolute errors for each category. None of the groups differ significantly on $\hbox {p} < .05$ (Supplementary Table 4). ^23^.

### Model performance vs. data heterogeneity

The assessment of the predictive performance by visual interpretation of 200 independent images per trait regarding the distance of the photographer to the target species (‘image-target distance’) showed that images with the longest distance attained 7.6%, whereas most images showed the shortest distance (63.5%; Fig. 4). Additionally, the vast majority of the images (92.9%) expressed a high image quality, meaning that all plant organs appearing in the image were clearly recognisable. The assessment of the growth form (woody vs. non-woody) of the images’ target species revealed that the share of woody and non-woody species was approximately equal. None of the groups in Fig. 4 differed significantly on $\hbox {p} < .05$ (Supplementary Table 4).

**Figure 5:**
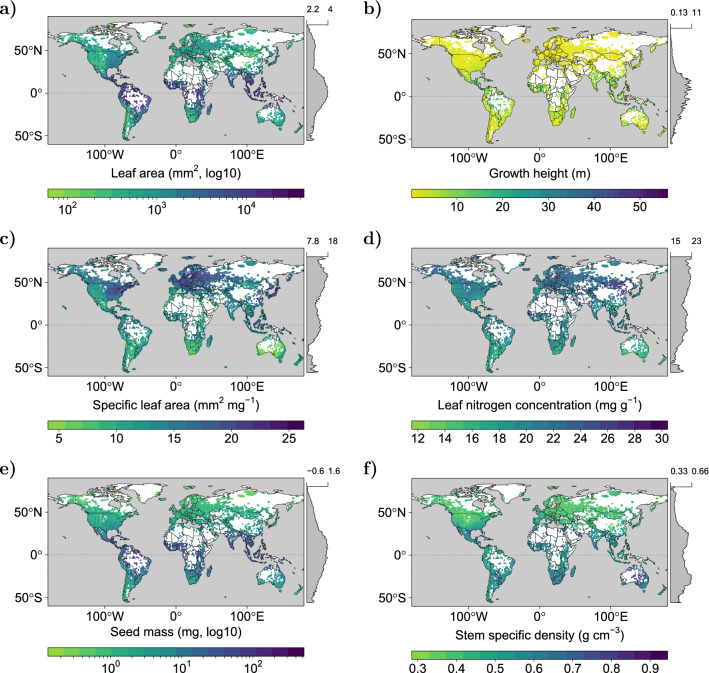
Global trait distribution maps. Global maps of mean plant functional traits produced by inverse-distance weighted interpolation on ensemble predictions for leaf area (**a**), growth height (**b**), specific leaf area (**c**), leaf nitrogen concentration (**d**), seed mass (**e**), stem specific density (**f**) including latitudinal distribution. Values of leaf area and seed mass were $\log _{10}$-transformed for improved visualisation.

### Global trait distribution maps and their validation

Global trait distribution maps (GTDM) derived from model predictions on more than 185,000 independent plant photographs, which were not used for model training, expressed a unimodal latitudinal distribution peaking around the equator for LA, GH and SM (Fig. 5). SLA and LNC expressed their highest values around northern temperate and polar zones as well as the equator. SSD showed a bimodal distribution with peaks in the subtropics. All trait expressions were similar along the equator. A gradient from high to low LA and SLA was found from the east of North America towards the west, while an opposite trend was observed for LNC and SSD. A quantitative comparison with published GTDM revealed significant correlations with Pearson’s $\hbox {r} > .5$ concerning GH^26^, SM^6^ and SSD^6,26^, whereas insignificant or small correlations were found between all of the four GTDM concerning LNC^26–28^ (Supplementary Fig. 1). For SLA, our GTDM correlated significantly to ref.^6,26^ ($\hbox {r} > .5$), whereas correlations were negative or insignificant between the former and ref.^27,28^.

## Discussion

Our results suggest that certain plant functional traits can be retrieved from simple RGB photographs. The key for this trait retrieval are deep learning-based Convolutional Neural Networks in concert with Big Data derived from open and citizen science projects. Although these models are subject to some noise, there is a wealth of applications for this approach, such as global trait maps, monitoring of trait shifts in time and the identification of large-scale ecological gradients. This way, the problem of limited data that still impedes us to picture global gradients^7^ could be alleviated by harnessing the exponentially growing iNaturalist database^16^. The performance of the CNN models across traits varied strongly, but revealed a clear trend: As expected, the more a trait referred to morphological features, the more accurate the predictions were. The models of the Baseline setup explained a substantial amount of the variance for LA and GH, whereas traits that are partly related to morphological features, SLA and SM, show moderate $R^2$ values. The predictions of LNC and SSD explain almost none of the variance, suggesting that tissue constituents are not directly expressed or related to visible features. It also indicates that the strong covariance among these traits^13^ does not suffice in supporting their predictions from photographs. If the RGB images do not contain relevant information, the model will minimise the prediction errors by the regression-to-the-mean bias seen in Fig. 3 (especially lower panels).

The value of informing the model on the known trait variability through an augmentation of the target values (Plasticity setup) depended on the results of the Baseline setup of the trait. That is, the better the predictive performance of the Baseline setup, the more the trait seemed to profit from the Plasticity setup, rendering it ineffective for SSD, LNC and SM (Fig. 3). Refraining to cling to species mean values by considering within-species trait variation has been applied before using conventional methods^26^, but to our knowledge has never been tried in CNN models, yet. We expected that providing a distribution of trait values rather than a single mean for each species can convey to the CNN that different trait realisations can be expected from the same species. Obviously, this idea can only work if a distribution rather than a single value is available for each species. The SM dataset, for instance, contained only one image per species (Table 1). In this case, the Plasticity setup reduced the predictive performance compared to the Baseline setup, possibly by increasing the discrepancy to the true trait value. Since the traits with more accurate predictions profited most from the Plasticity setup, we assume that it supports the model in learning to predict the trait expressions themselves rather than extracting them indirectly through taxa-specific morphological features. Given that we restricted the number of images per species to a maximum of 8, while successful deep learning-based plant species identification usually requires thousands of images^12,22^, it seems very unlikely that the models inferred traits from species-specific plant features visible in the imagery. The latter was underpinned by our finding that the predictions of most traits are void of phylogenetic autocorrelation (Supplementary Information 1 and Supplementary Table 5), indicating that taxonomic relationships were insignificant for the trait predictions. The absence of phylogenetic autocorrelation of the prediction errors underlines that the models did not learn species-specific features for most traits, as this would imply similar trait predictions for related species.

On the contrary, the SSD model predictions express a phylogenetic signal (Supplementary Information 1 and Supplementary Table 5). Trait expressions are generally clustered under similar climatic conditions^29–31^. Simultaneously, climatic conditions constrain the geographic distribution of species and growth forms^29–32^. The SSD dataset is biased towards woody species (Table 1), which confines it to a smaller taxonomic range. Hence, the phylogenetic signal of SSD might result from its phylogenetic clustering and predominant dependence on bioclimatic information rather than on RGB imagery (Fig. 2).

Nevertheless, the benefit of including climate information on temperature, precipitation and their seasonality^8,20,21,26^ on predicting trait expressions was confirmed for all traits in this study, which underlines the value of contextual constraints in CNN models^10^ (see below for a discussion of the relevance of climate vs. image data). This also highlights the general flexibility of deep learning frameworks in adapting to variable input data from different scales and sensors^10^, which makes them a promising tool for ecological research. Our results particularly revealed this effect for SSD, SLA and LA, whereas it was smaller for GH, LNC and SM (Fig. 2). For the latter traits, other physical constraints such as disturbance^33,34^, seasonal variation^35,36^ and soil conditions^6,26,28^ come into consideration. As the focus of the Worldclim setup was to show that contextual cues can improve the trait retrieval from photographs rather than identifying the best set of auxiliary data, we confined the analysis to the most promising^20,26^ data source (WorldClim^37^).

In the Worldclim setup, a single model accumulated knowledge about the trait learning task. Combined predictions of different CNN models, however, have shown to surpass the predictive performance of single CNN, e.g. in plant species identification tasks^22^. Each of the CNN models is prone to literally ‘look’ at different aspects of the learning task by focusing on different image features. Previous research also showed increased model performance in a trait prediction task in case of ensembles of regression and machine learning models^26^. Accordingly, and as demonstrated by our results, an ensemble approach seems promising to further enhance predictive performance of CNN models concerning trait prediction.

The predictive performance of these Ensembles has shown to be reproducible with different sets of training images (cp. Figs. 2, 3, Supplementary Fig. 2). In our heterogeneous dataset, model performance was not affected by different growth forms, image qualities and image-target distances (Fig. 4). Different growth forms and plant functional types show their own characteristic trait spectrum^13^. Possibly, contextual cues within the image might have supported the CNN in inferring the plant functional type of a species, e.g. by a long-distance image being indicative of a tree species. Yet, since the majority of the images only shows single plant organs on close-up photographs (Fig. 4), we assume that the trait predictions are not confounded by the identification of growth forms. Furthermore, the absence of a phylogenetic signal in the prediction errors for most traits highlights the model’s ability to generalise by extracting trait information independent of taxonomic relationships, meaning that the models (except for SSD) did not learn species-specific mean trait expressions (see Supplementary Information 1 and Supplementary Table 5).

Additionally, we disclosed the high generalisability of these results by investigating the datasets’ underlying distributions both spatially (Supplementary Fig. 3) and across biomes (Supplementary Fig. 4). Although some regions such as Central Europe and North America show higher data coverage, the datasets used for this study contain data across all biomes and regions on Earth. Therefore and despite this clustering, we expect the models to be applicable for all biomes around the globe. This was highlighted by an additional analysis showing that the predictive performance of the models is reasonably constant across biomes (Supplementary Fig. 5). As suggested by refs.^38,39^, we tolerated a certain spatial bias in favor of larger datasets. Although the SSD dataset predominantly contained woody species, neither of the six datasets expressed an exclusion of either growth form (Table 1, Fig. 4).

The application of our models to global gradients of traits revealed that our GTDM indeed cover macroecological patterns and trends known from other publications: The latitudinal distributions could roughly be confirmed for GH^26^, LNC^26,27^ and SM^6,8^ (Fig. 5). Predicted trends for maximum leaf size hint at the applicability of our GTDM of LA^40^. The trait gradients for North America were confirmed for SLA^6,8,26–28^, SM^6,8^, LNC^27^ and SSD^6^ alike. Although based on different input data and modelling methods, the major global latitudinal gradients found in previous studies could be reproduced by our GTDM, which indicates the plausibility of the latter^6,8,26,27^.

We further validated the GTDM quantitatively by means of correlations with other GTDM. Regarding SSD, the detected high correlations might be due to method similarity, as our GTDM product of SSD primarily builds upon climate data (see above), just as ref.^6,26^. For GH, SLA and SM, however, the high correlations are unlikely to result from climate data exclusively, as the explained variances of the RGB imagery ($R^2$ of Plasticity setup) are higher than the additional contributions of the Worldclim setup (approx. 94%, 70% and 79% share of imagery on total explained variance, respectively; Supplementary Table 2). We decoupled the GTDM products from bioclimatic information in an additional analysis (Supplementary Fig. 6). Remarkably, the macroecological patterns could roughly be reproduced when the GTDM were based exclusively on RGB imagery, which shows that the bioclimatic information merely serves to smooth the macroecological trait patterns for most of the traits.

Despite of all GTDM being at least partly build upon climate data and using trait data from the same source (TRY database), some GTDM of SLA and all GTDM of LNC vary strongly in their correlations (Supplementary Fig. 1). On the one hand, this might indicate that LNC varies at a different scale, e.g. on account of its seasonal and within-species variation^35,36^. On the other hand, other GTDM products are based on mean trait values weighted by abundances of plant functional types^27,28^ rather than single trait predictions, which might explain negative or non-significant correlations as well.

Hence, a potential pitfall of the presented approach is that it is prone to express an observation bias, e.g. by citizen scientists only taking pictures of the most striking species. The sampling design underlying the GTDM does not account for plant community composition, meaning that we cannot tell if plant photographs at a certain location represent the actual community structure. Since many images contain more than one individual plant and different species, the CNN model predictions, however, might be based on more than one species, thereby partly resembling trait expressions of the community. The representativeness of trait data for plant communities, though, remains an ubiquitous problem of global trait maps, including those fully based on trait data from the TRY database^7^, since every available dataset is far from representing the actual plant community composition^7^. Hence, at present our GTDM have to be considered a plausibility check of the model predictions rather than an application-ready trait map product, not least because the sampling of images might not be representative of the respective plant community.

Nevertheless, our results indicate that exploiting a Big Data approach is viable to reveal macroecological trait patterns, maybe because the most striking species of an ecosystem are likely to suffice in describing its functional footprint^5^. Since the strong growth of the iNaturalist database leads to a steadily increasing geographic coverage, the representativeness of these data is likely to grow as well. A recent study investigating the records of FloraIncognita^12^, a citizen-science and deep learning-based application for identifying plant species from photographs, suggested that such crowd-sourced data can reproduce primary dimensions in plant community composition^41^. This underlines the future potential of harnessing citizen science databases for identifying these patterns. Here, we demonstrated the practical value and applicability of the CNN models by producing GTDM that were able to reproduce known macroecological trait patterns while displaying one anticipated application of this method. Additionally, in these GTDM we bypass the issue of spatial error analysis that is challenging for most GTDM products^26^ by obtaining a potentially arbitrary number of observations in light of the strongly increasing number of observations in iNaturalist, almost rendering an extrapolation obsolete. Our GTDM are based on individual trait measurements rather than estimated on behalf of a small set of covariates, which is typical for climate-based GTDM^26^. Since plant traits vary strongly within species^17–19^, these measurements express a high practical relevance. As the iNaturalist plant photograph database is witnessing an exponential growth of data inputs, the potential of exploiting this data source for plant trait predictions is growing rapidly. It is worth mentioning that this approach also led to the first publication of a GTDM of mean LA (available for download under https://doi.org/10.6084/m9.figshare.13312040), since former publications were limited to modelling upper limits of LA based on climatic constraints^40^.

Future studies building on our work, which benefit from the ever-growing data accumulation of both the iNaturalist and TRY database, might not face restrictions of dataset size as we did in our study. This might allow for more representative samples in future studies, e.g. enabling to stratify training data by species while simultaneously balancing the trait distribution. This might support a reduction of the regression-to-the-mean bias seen in all of the results (Fig. 3) by avoiding to overrepresent common trait expressions. Another possible approach would be to select only species with particularly low variability for model training, since it decreases the chance of incorporating images showing plants with an extreme trait expression that differ strongly from the chosen mean trait values from TRY. By that, we might be able to derive more reliable and accurate predictions in the context of weakly supervised learning by reducing noise in the training data.

Although weakly supervised learning approaches generally have shown to be an effective way of compensating a shortage of individually labeled data^42,43^, an image dataset including in-field trait measurements under natural conditions representing the global trait spectrum would be necessary for a conclusive validation. In our study, it even remains unclear to what extent the trait values actually refer to the individual plant shown in an image, particularly as the images sometimes show more than one individual plant and more than one species (Supplementary Fig. 7). This may hinder the model from predicting a trait value corresponding to the dominant species in the image (but might also partly resemble the community composition, see above). Although we attempted to compensate the lack of a dataset that enables a conclusive validation by eliminating possible biases concerning image settings (Fig. 4), growth forms (Fig. 4), phylogenetic autocorrelation (Supplementary Information 1, Supplementary Table 5), predictions based only on climate data (Supplementary Fig. 6), predictive performance across biomes (Supplementary Fig. 5), a training dataset subject to limited geographic or climatic coverage (Supplementary Figs. 3, 4) and effects of a specific set of training data (Supplementary Fig. 2), we cannot conclusively prove that the model predictions are based on causal relationships. Our model results suggest that the trait predictions reflect the feature space of natural trait expressions (Fig. 3), but an in-depth analysis of the image features the models learned for inferring the respective traits will be necessary to rule out any possible biases in future studies. An explicit analysis might involve investigating which plant organs are relevant for the trait predictions by means of feature attribution techniques and could ultimately provide clear evidence. This may not only enable to build trust in such artificial intelligence (AI) models, but also to generate new knowledge from them in order to deepen our understanding of plant morphology and trait covariance.

Nevertheless, this study can only be considered a pioneering work testing the feasibility of the approach, as application-ready models require a conclusive and explicit validation. A dataset enabling this has to incorporate image-trait pairs measured and photographed on the same individuals. One possible solution would be to generate a database of plant traits including respective photographs, which then can serve as a benchmark for future studies.

## Conclusion and outlook

Following the urgently needed transition of ecology towards a data-sharing scientific discipline^15,44^ and the call for integration of powerful datasets in ecology^44^, we built upon this revolution of Big Data provided by professional and citizen science alike. Therefore, we exploited the potential of Convolutional Neural Networks^44^ to produce generic models generalising across all biomes and regions around the globe to extract a set of plant functional traits from simple RGB photographs. Thus, the burden of limited geographical coverage of trait databases might be lifted by the worldwide coverage of the iNaturalist plant photograph database. The traits referring to the models with the highest predictive performance cover the primary axes of plant form and function ^13^, which are plant and organ size (GH, LA) as well as the leaf economics spectrum (SLA). Despite of the disputable model performance for some traits, our results highlight the potential of this approach to facilitate non-invasive, cost-efficient and automated assessments of functional gradients in future real-world applications. In order to achieve application-ready models, however, a conclusive validation is mandatory and has to incorporate image-trait pairs derived from the same individual plants. Once this is done, future applications of this approach might be next generation global trait maps^33,34^ as input for modelling and monitoring ecosystem processes and biochemical cycles, trait monitoring on time series data from PhenoCam images^45^ on local scales, or assessments of ecosystem functions on landscape-scale through high resolution imagery from drones^11^. Each of those applications might empower researchers to further close the gap between actual and intended spatio-temporal coverage, which ecology has been falling short of for decades^46^.

## Methods

### Data acquisition and preparation

We acquired trait records of the six plant functional traits leaf area (LA), growth height (GH), specific leaf area (SLA), leaf nitrogen concentration (LNC), seed mass (SM) and stem specific density (SSD) from the TRY database^7^ (see Supplementary Table 1 for details). These traits explain most of the global trait variation in plants^13^ since they directly relate to plant’s nutrient economics and competitive abilities^3,4,13^. Accordingly, these traits are among those with the highest data coverage in the TRY database^7^. The traits LA and GH could be readily visible by computer vision, whereas traits such as SLA and LNC might be predictable through visible plant features such as thickness, color intensity or shape of leaves. Moreover there is a strong covariance between all of these traits, for instance a high GH usually implying a high SSD^3,13^. We utilised the standardized values given in the TRY database, which have been converted to uniform units. Observations with a difference to the trait mean value of greater than 4 were removed as recommended in the TRY release notes, since these observations are considered outliers. Next, the mean and standard deviation was computed for the six plant traits for each species over all observations.

Plant photographs together with their geolocation were downloaded from the iNaturalist database (Research-grade Observations) via the Global Biodiversity Information Facility (GBIF)^47^. The observations containing presumed wrong species names and geospatial issues such as missing coordinates and a coordinate uncertainty of more than 100 km were removed from analysis. Next, the geolocations from the photographs’ metadata were used to extract bioclimatic variables from the Worldclim database with a resolution of $0{^{\circ }}$ 10’ 0”^37^. We chose the following bioclimatic variables: Annual Mean Temperature (BIO1), Temperature Seasonality (BIO4), Temperature Annual Range (BIO7), Annual Precipitation (BIO12) and Precipitation Seasonality (BIO15). Additionally, we computed the Precipitation Annual Range (BIO13-14) by subtracting Precipitation of Wettest Month (BIO13) by Precipitation of Driest Month (BIO14). BIO1 and BIO12 are known predictors of plant traits^6,17,20,21^. Similarly, climate variables referring to range and seasonality have shown to coincide with plant traits^8,26^. Therefore, we selected the other four climate variables (BIO4, BIO7, BIO13-14, BIO15) to characterize annual variation of climatic conditions of the respective site. Photographs that could not be linked to climate data, e.g. because of geolocations off the land surface, were removed from the analysis.

### Sampling and pre-processing

Based on the species names, we linked the trait observations obtained from the TRY database (species-specific mean and standard deviation) with the plant photographs (iNaturalist) already linked to bioclimatic variables (Wordclim). For the purpose of balancing the dataset, i.e. avoiding overrepresented species, while maximizing the overall dataset size, we included at least one but at maximum eight observations per species (Table 1). We sampled a wide range of species rather than focusing on an equal distribution of trait expressions, since we wanted to prevent the model from learning species-specific trait expressions. Therefore, the number of images per species was kept as low as possible, and the number of species in the dataset as high as possible, while attempting to achieve a sufficiently large dataset of at least 10,000 images. Next, we extracted a random sample of 10% of the dataset of each trait before model training. This ‘test dataset’ was not involved in the training process and exclusively served for the independent evaluation of the trained models. The remaining data was split into ’training dataset’ and ’validation dataset’ by a ratio of 4:1 (Table 1). The training dataset was employed to train the weights of the CNN model, whereas the validation dataset indicated the training progress after each full training cycle (’epoch’).

We clipped the images to be quadratic by removing the spare margins and down-sampled the resulting image to a resolution of 512 × 512 pixels. Further pre-processing included $\log _{10}$-transformation of the reference trait data (‘targets’) due to skewed distributions and removing outliers exceeding three standard deviations above or below the mean. Afterwards, we normalised all targets (training, validation and test datasets) by the minimum and maximum values of the training dataset according to

1 $$\begin{aligned} target_{norm} = \frac{target - min_{train}}{max_{train} - min_{train}}, \end{aligned}$$

target denoting the $\log _{10}$-transformed target value, and $\mathrm {min}_{\mathrm {train}}$ and $\mathrm {max}_{\mathrm {train}}$ being the minimum and maximum of the $\log _{10}$-transformed training dataset. Note that the minimum and maximum values used for normalising the targets were derived from the training dataset exclusively, preventing a leakage of information of the validation and test datasets to the training process. As a result, the final target values ranged exactly (for validation and test datasets: approximately) between 0 and 1. The same normalisation scheme was undergone for the bioclimatic variables.

### Convolutional neural network setups

CNN, a sub-group of deep learning models for image analysis, are designed to harness the spatial context of image pixels for a specific modelling problem. The basic structure of CNNs includes a cascade of convolutions, i.e. optimizable filter operations, for extracting activation maps and down-sampling (’pooling’) operations, which enable to perform these operations at multiple spatial scales. Hence, the feature maps derived this way can contain both low- (e.g. edges) and high-level features (e.g. leaf shapes) of the image. Depending on the target value, the CNN learns which features are relevant and aggregates this information to a specific prediction in the last layer. In general, CNNs are computationally efficient and do not require a manual feature design process, making them readily adaptable to many image interpretation tasks. Former studies revealed their applicability in vegetation science tasks such as plant species identification using plant images^12,22,48^ and identification of plant communities utilising imagery from unmanned aerial vehicles (UAV)^11^. In view of our research objectives, we tested the potential of CNN for plant trait retrieval with four different setups (Fig. 1):

1. As a baseline, a state-of-the-art CNN architecture called Inception-Resnet-v2^49^ was used (’Baseline’ in Fig. 2, setup 1 in Fig. 1).
2. In order to test if prior knowledge on within-species variability can improve the weakly supervised learning process, ’Plasticity’ (or ’target augmentation’, TA) was implemented with exactly the same model configuration as in setup 1) (’Plasticitiy’ in Fig. 2, setup 2 in Fig. 1). Plasticity was realised by harnessing the standard deviations obtained from TRY database regarding every trait and species (for information on data availability, see Table 1). The target values were altered within a Gaussian distribution truncated by one standard deviation of the trait values using R package ’truncnorm’ (version 1.0–8)^50^, thereby leaving a small deviation from the mean value more likely than a large one. To avoid negative as well as overly large values, the targets were clipped to the range between 0 and 1 after normalisation.
3. As a contextual constraint, bioclimatic data (see above) was fed into the CNN with the same model and data configuration including Plasticity in a mixed data approach (‘Worldclim’ in Fig. 2, setup 3 in Fig. 1). 4) We tested an Ensemble approach, in which two more state-of-the-art model architectures, namely Xception^51^ and MobileNetV2, the latter with halved number of trainable parameters^52^, were trained on the configuration of setup 3, and their predictions were subsequently averaged (’Ensemble’ in Fig. 2, setup 4 in Fig. 1). These models differed strongly in their number of trainable weights, resulting in a different depth. The final model performance was assessed using a 3-fold cross-validation, with three different training, validation and test splits. To enable a comparison of model performance across traits, the resulting mean absolute error (MAE) was normalised by division over the range of the target values of the respective test dataset (’normalised mean absolute error’, NMAE).

### Training process and hyperparameters

In order to build upon a pre-existing knowledge base, we employed ’transfer learning’ by using pre-trained layer weights (the storage of the model’s knowledge) from a classification task on a dataset on www.image-net.org^38^ for all CNN models used in this study. The regressor following the basic CNN consisted of a global average pooling layer followed by two dense layers with 512 and 1 output units. The latter forces the CNN to output exactly one prediction (trait) value. In case of the mixed data model (setups (3) and (4)), the CNN consisted of parallel branches to incorporate the different input data types. The branch processing the bioclimatic data consisted of three dense layers with 64, 32 and 4 output units, and the last layer of the CNN regressor contained 4 output units. After concatenating the two branches (image and bioclimatic branch), the regressor contained four dense layers with 8, 8, 4 and 1 output units. The last dense layer of each branch and the final layer of the model were linearly activated, whereas for all other dense layers, a ’relu’ activation function was utilised. The latter enables the model to use non-linear separation boundaries of the feature space. We determined this configuration by its model performance in preliminary runs.

We increased the robustness and transferability of the model predictions by means of ’image augmentation’ (also: ’data augmentation’), which works independent of the choice of the specific CNN architecture^53^. The image augmentation procedure serves to inflate the amount of training data, while simultaneously assisting the CNN to learn spatial features independent of the data acquisition, e.g. camera settings. Therefore, the images of the training data were subjected to horizontal and vertical flipping as well as adjusting the contrast, saturation and brightness (between factors of .9 and 1.1 each) of the images. After these adjustments, the pixel values were clipped to a range between 0 and 1 in order to prevent the value range from being enlarged.

For the training process, a batch size of 20 images and an RMSprop optimiser with a learning rate of 0.001 and a learning rate decay of 0.0001 was used. The chosen loss function was mean squared error, while the prediction accuracy was quantified by the MAE of the respective dataset. The MAE of the validation dataset was computed after each epoch. Models were trained until the validation MAE did not further improve compared to the preceding epochs and diverged from the training MAE (’overfit’). The trained model was then applied to the test dataset.

All CNN were implemented using the Keras API version (2.3.0.0)^54^ and the TensorFlow backend (version 2.2.0)^55^ in R (version 3.6.3)^23^. Model training was undergone on a workstation with two CUDA-compatible NVIDIA GPUs (GeForce RTX 2080 Ti, CUDA version 11.0).

### Evaluation

We tested the robustness of the CNN models in view of the heterogeneous dataset on 200 images per trait retained before training. Therefore, we extracted three criteria for each image: 1) We allocated the growth form (woody vs. non-woody) to the image using information from the TRY database. 2) The first author assessed the image quality in three categories (low, medium and high) as well as 3) the distance of the photographer to the target species in the image (’image-target distance’, categories: $<1\hbox {m}$, 1–5m and $>5\hbox {m}$) by visual interpretation (see Supplementary Information 2 for details and Supplementary Fig. 7 for example images). We predicted the respective trait values for these images using the trained Ensemble models and tested for significant differences of the MAEs across the three criteria above.

### Global trait distribution maps

We acquired further images including geolocations from the iNaturalist database via GBIF (see above) with a stratified sampling design, attempting to get the most even distribution possible by countries on Earth. Pre-processing, including bioclimatic data, was undergone as described above. We removed duplicates with the training and validation datasets of the respective trait in order to ensure that all of the images were unknown to the CNN model to avoid artefacts from the training process. This resulted in 188,156 (LA), 188,318 (GH), 186,873 (SLA), 187,115 (LNC), 188,523 (SM) and 185,688 (SSD) images. Next, we employed the trained Ensemble models to predict each of the six traits. Afterwards, we re-transformed the predictions to yield the traits in their original unit by reconverting Eq. (1). Then, we applied an inverse-distance weighting interpolation for each trait on all of the predictions using their geolocations. The result was averaged for each cell of a grid with a resolution of $0{^{\circ }}$ 30’ 0” that was superimposed on the world map in WGS84 coordinate reference system using the R package ’raster’ (version 3.3-13)^56^. To minimize uncertainties associated with extensive extrapolations^26^, we masked the interpolation output with a buffer of 100 km around each observation. Additionally, we excluded all grid cells that did not fall within the Earth’s landmass. For the same grid cells, the .9 and .1 quantile was computed and their difference was mapped as the quantile range for reference (see Supplementary Fig. 8). All trait maps were produced with R package ’rasterVis’ (version 0.49)^25^.

### Quantitative plausibility check of global trait distribution maps

We obtained the GTDM from ref.^6,26–28^ in order to check the plausibility of our GTDM. All maps had the same coordinate reference system (WGS84), but were resampled to the same grid as our maps by bilinear interpolation using R package ’raster’ (version 3.3-13)^56^. The Pearson correlation coefficients and their significance value was computed for all available GTDM combinations and plotted using the R package ’corrplot’ (version 0.84)^57^.

## Supplementary Information

Supplementary Information.

## Data Availability

The image data (Fig. 5 and Supplementary Fig. 8) and all CNN models of the Ensemble setup supporting the findings of this study are available in ’figshare’ with the identifier https://doi.org/10.6084/m9.figshare.13312040. Additionally, the raw data tables containing the download links for the plant images as well as the mean trait values that were the basis for further processing are available on figshare (https://doi.org/10.6084/m9.figshare.14410379). The raw image dataset can be obtained from iNaturalist database via https://doi.org/10.15468/ab3s5x^47^. Raw trait data are available upon request from the TRY database (https://www.try-db.org/)^7^.

## Code availability

The code supporting this manuscript is available online at https://github.com/ChrSchiller/cnn_traits.
