# Supplementary material for: Deep learning and citizen science enable automated plant trait predictions from photographs
Source: Sci Rep. 2021 Aug 12;11:16395. doi: 10.1038/s41598-021-95616-0 (PMC8361087; doi:10.1038/s41598-021-95616-0)
Supplement: Supplementary file 1 — Supplementary Information. [file 41598_2021_95616_MOESM1_ESM.pdf]

Deep Learning and Citizen Science Enable Automated Plant Trait Predictions from Photographs - Supplementary Material

[1]Christopher Schiller [1]Sebastian Schmidtlein [2]Coline Boonman [3]Alvaro Moreno-Martínez [4,*]Teja Kattenborn

[1] Institute of Geography and Geoecology, Karlsruhe Institute of Technology (KIT), 76131 Karlsruhe, Germany

[2] Department of Environmental Science, Institute for Water and Wetland Research, Radboud University, Nijmegen, the Netherlands

[3] Image Processing Laboratory (IPL), Universitat de València, València, Spain

[4] Remote Sensing Centre for Earth System Research, Leipzig University & Helmholtz Centre for Environmental Research – UFZ, Leipzig, Germany, 04103 Leipzig, Germany

[*] teja_kattenborn@uni-leipzig.de

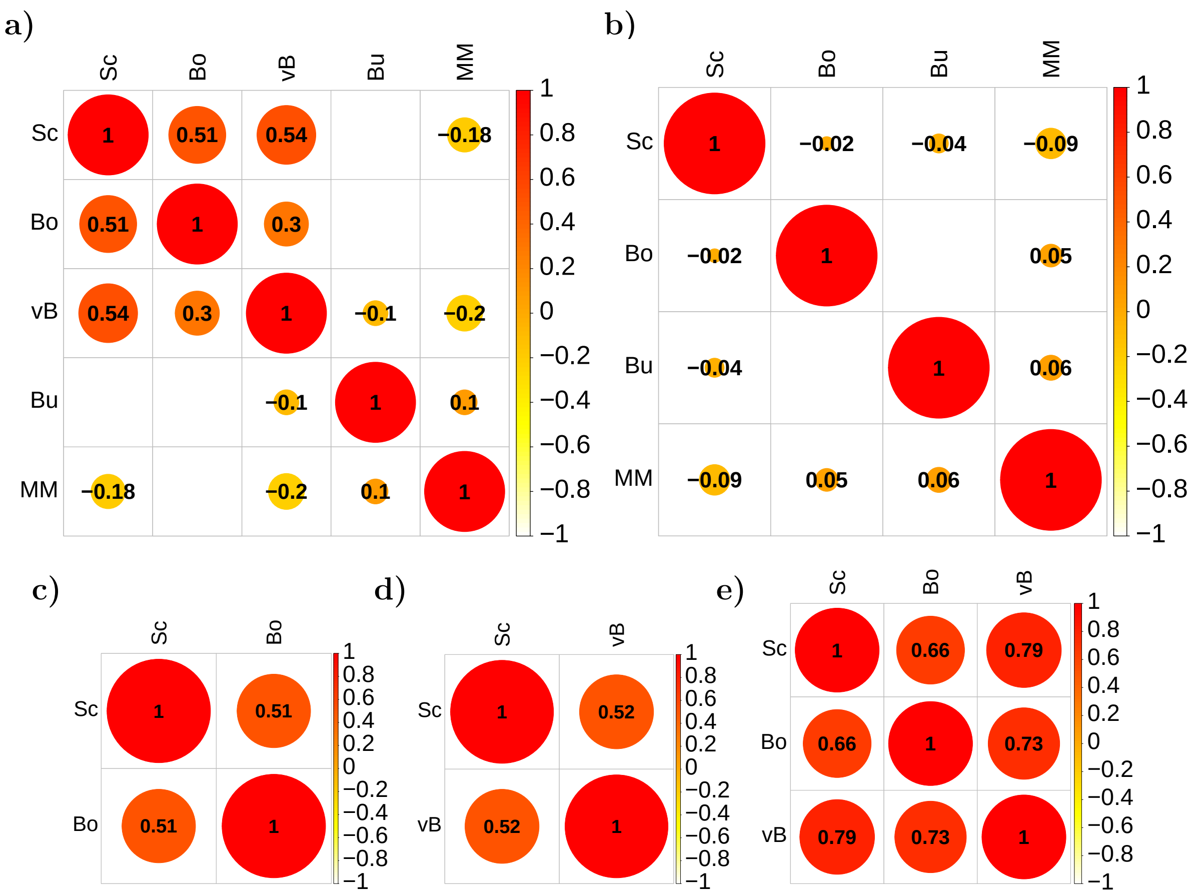

Supplementary Figure 1: Correlation plots comparing global trait distribution maps. a-e, Pearson correlation coefficients between our work (Sc), ref.[1] (Bo), ref.[2] (vB), ref.[3] (Bu) and ref.[4] (MM) concerning specific leaf area (**a**), leaf nitrogen concentration (**b**), growth height (**c**), seed mass (**d**) and stem specific density (**e**). Color scheme and size of circles indicate direction and strength of correlation. Only significant correlations of $p < .05$ are shown.

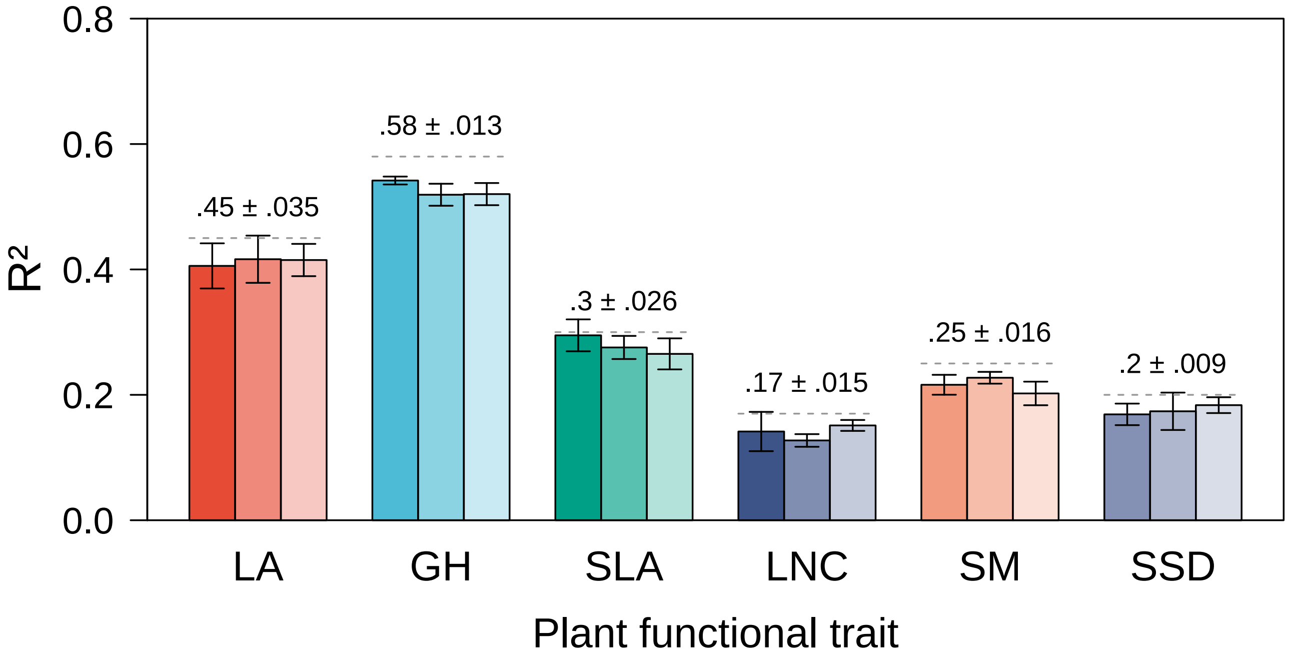

Supplementary Figure 2: Details on cross-validation results. Explained variance (R^2) and standard deviations for each of the three CNN models included in the Ensemble setup of 3-fold cross-validation for the plant functional traits leaf area (LA), growth height (GH), specific leaf area (SLA), leaf nitrogen concentration (LNC), seed mass (SM) and stem specific density (SSD). CNN architectures used in the model runs were Inception-Resnet-v2 (left bar for each trait), Xception (center bar), MobileNetV2 (right bar). Dashed grey line and figures above them indicate Ensemble result including standard deviation. This figure highlights small standard deviations across different training-test splits, which indicates that the model results are robust across different training images.

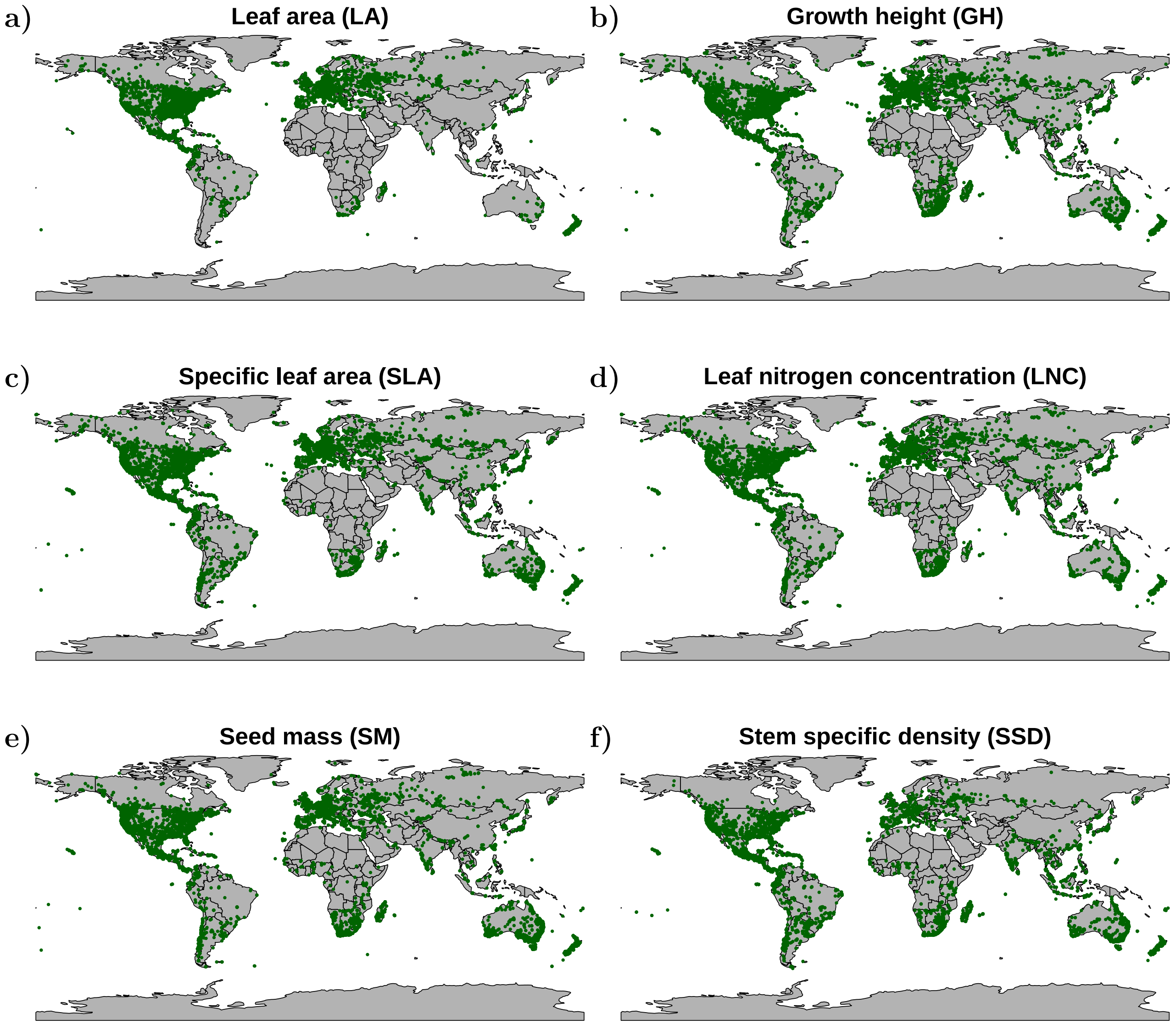

Supplementary Figure 3: Geographic coverage of the image-trait datasets. a-f, Green points, geographical occurrence of image records for datasets concerning leaf area (**a**), growth height (**b**), specific leaf area (**c**), leaf nitrogen concentration (**d**), seed mass (**e**) and stem specific density (**f**). WGS84 coordinate reference system; grey, land surface.

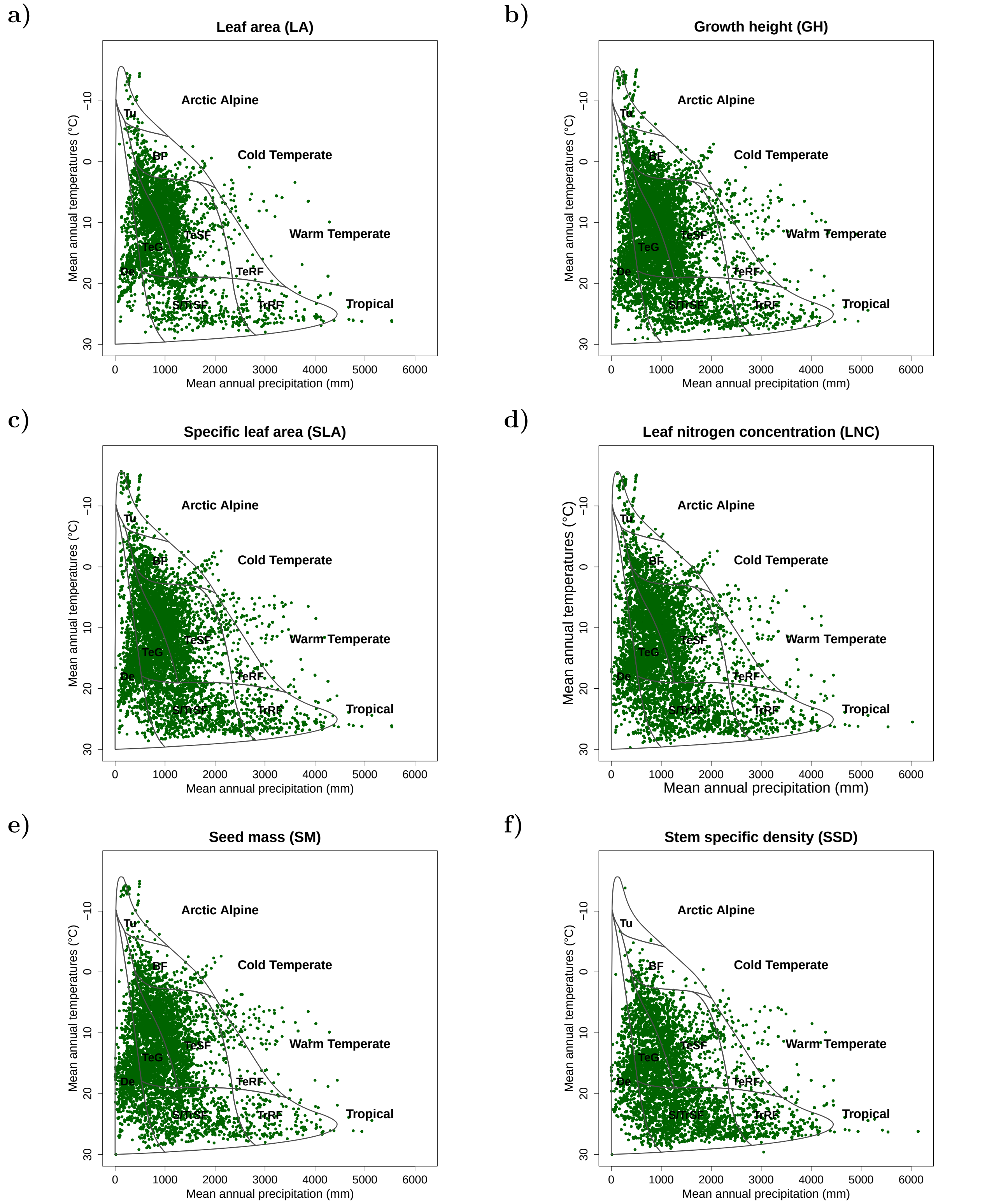

Supplementary Figure 4: Climatic coverage of the image-trait datasets. **a-f**, Distributions indicating the mean annual temperature in °C and mean annual precipitation in mm for the geolocations of the image records of the datasets for leaf area (**a**), growth height (**b**), specific leaf area (**c**), leaf nitrogen concentration (**d**), seed mass (**e**), and stem specific density (**f**). Tu, tundra; BF, boreal forest; De, desert; TeG, temperate grassland; TeSF, temperate seasonal forest; TeRF, temperate rain forest; S/TrSF, savannah and tropical seasonal forest; TrRF, tropical rain forest. Biome boundaries are based on the R package 'plotbiomes' (version 0.0.0.9001), accessed via https://github.com/valentinitnelav/plotbiomes on 2020-10-10.

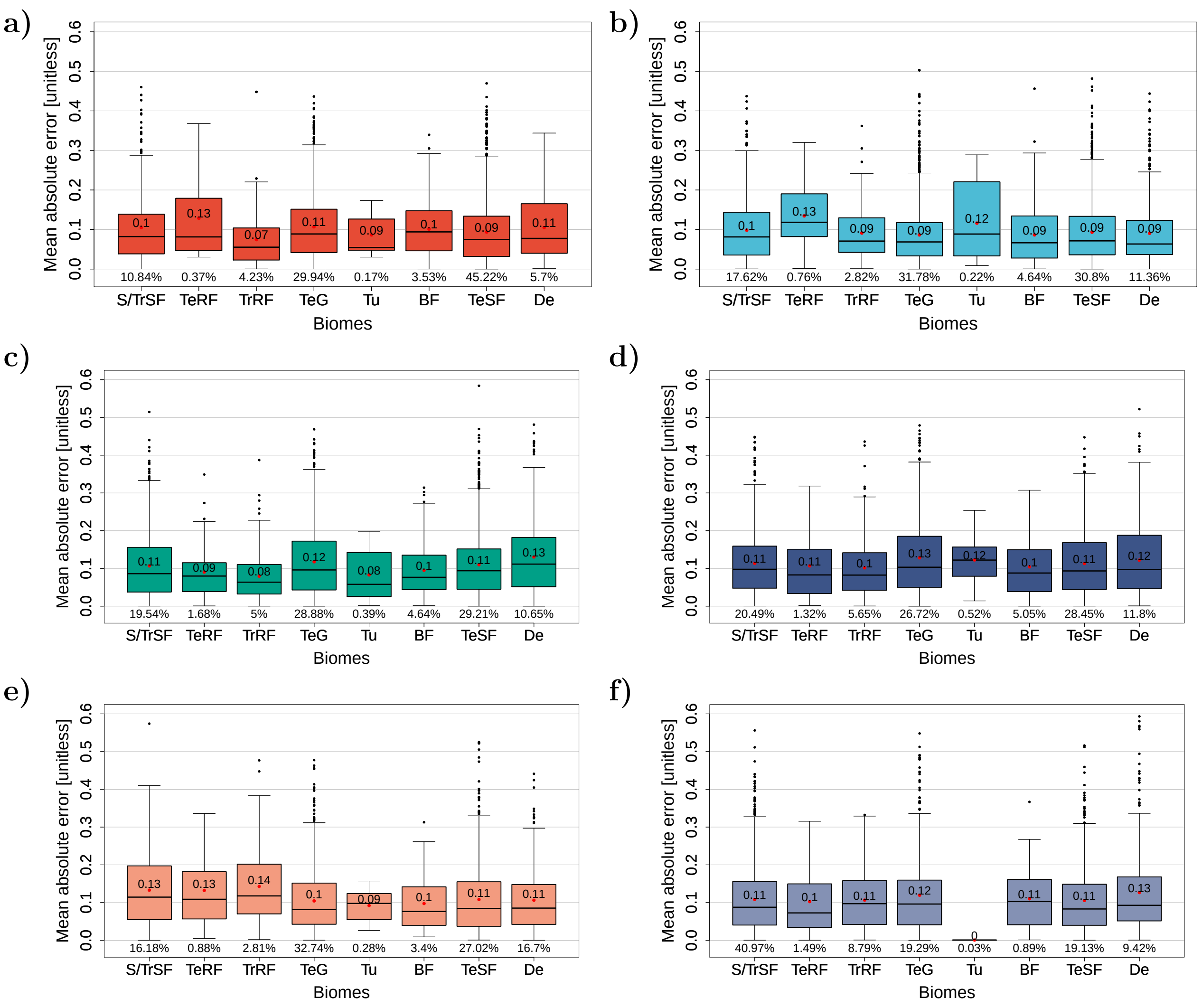

Supplementary Figure 5: Model predictive performance across biomes. **a-f**, Boxplots indicating the mean absolute error across biomes for the test datasets of the 3-fold cross-validation for leaf area (**a**), growth height (**b**), specific leaf area (**c**), leaf nitrogen concentration (**d**), seed mass (**e**), and stem specific density (**f**). The percentage of data points for each biome is indicated below each box plot. Red dots and the number linked to them are the mean values for each category. S/TrSF, savannah and tropical seasonal forest; TeRF, temperate rain forest; TrRF, tropical rain forest; TeG, temperate grassland; Tu, tundra; BF, boreal forest; TeSF, temperate seasonal forest; De, desert.

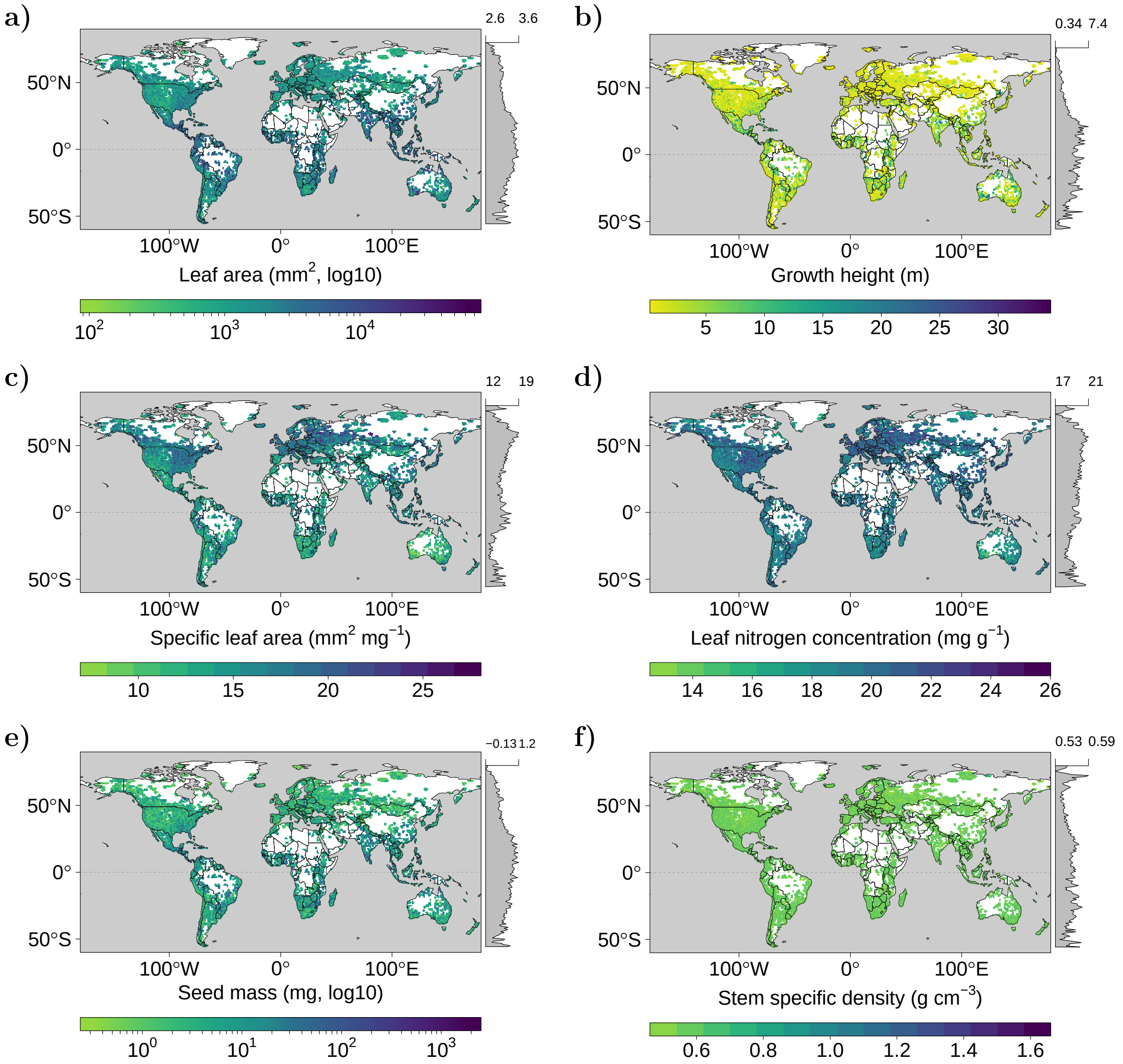

Supplementary Figure 6: Global trait distribution maps without bioclimatic variables. Maps of mean plant functional traits produced by inverse-distance weighted interpolation on trait predictions for leaf area (**a**), growth height (**b**), specific leaf area (**c**), leaf nitrogen concentration (**d**), seed mass (**e**), stem specific density (**f**) including latitudinal distribution using Plasticity setup, thereby omitting the implementation of bioclimatic variables. Values of leaf area and seed mass were $\log_{10}$-transformed for improved visualisation.

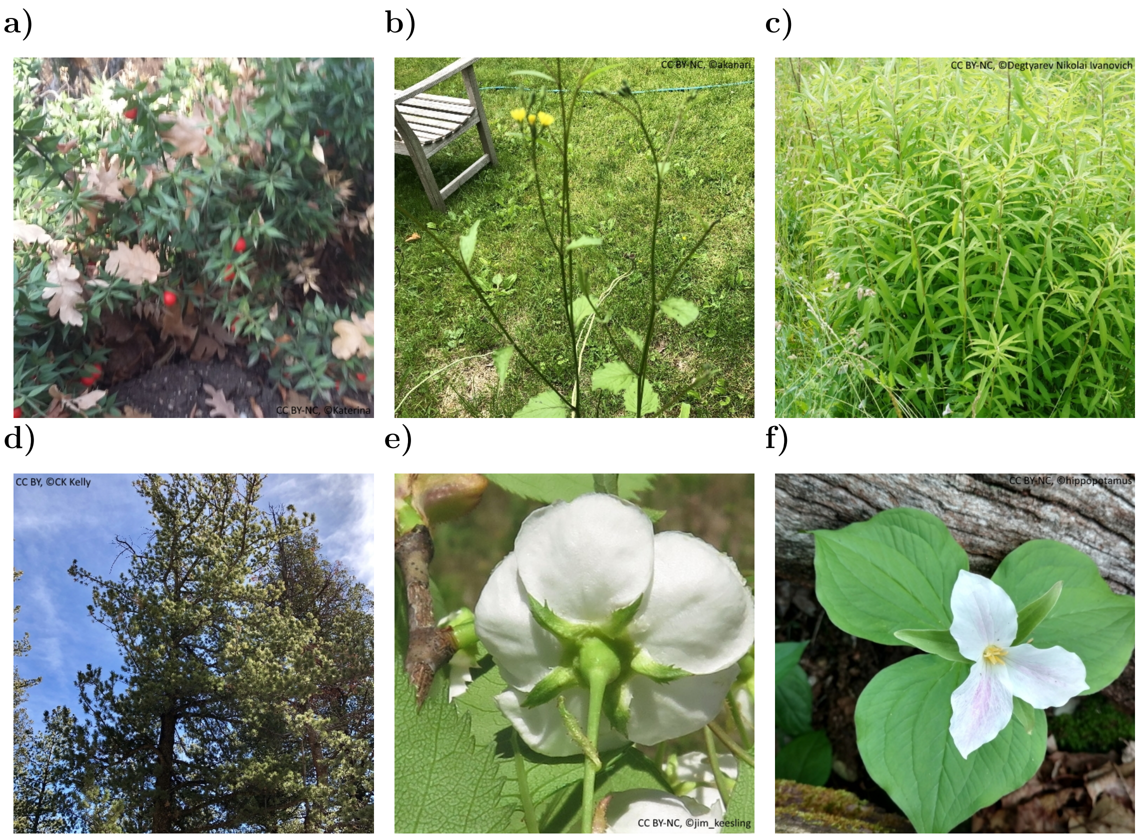

Supplementary Figure 7: Example photographs. a-f, Examples for distance category < 1 m (**e, f**), between 1 and 5 m (**a-c**), and > 5 m (**d**) as well as quality category low (**a**), medium (**b**) and high (**c-f**). Images are available under Public License (indicated in the images including rightsholder). Image URL's (last accessed on 2020-10-21):
(a) https://static.inaturalist.org/photos/56573588/original.jpg?1574145100,
(b) https://static.inaturalist.org/photos/41554312/original.jpg?1560179435,
(c) https://static.inaturalist.org/photos/62153756/original.jpeg?1582609829,
(d) https://static.inaturalist.org/photos/29219713/original.jpg?1544973895,
(e) https://static.inaturalist.org/photos/53871278/original.jpeg?1570887169,
(f) https://static.inaturalist.org/photos/37947883/original.jpeg?1557005441.

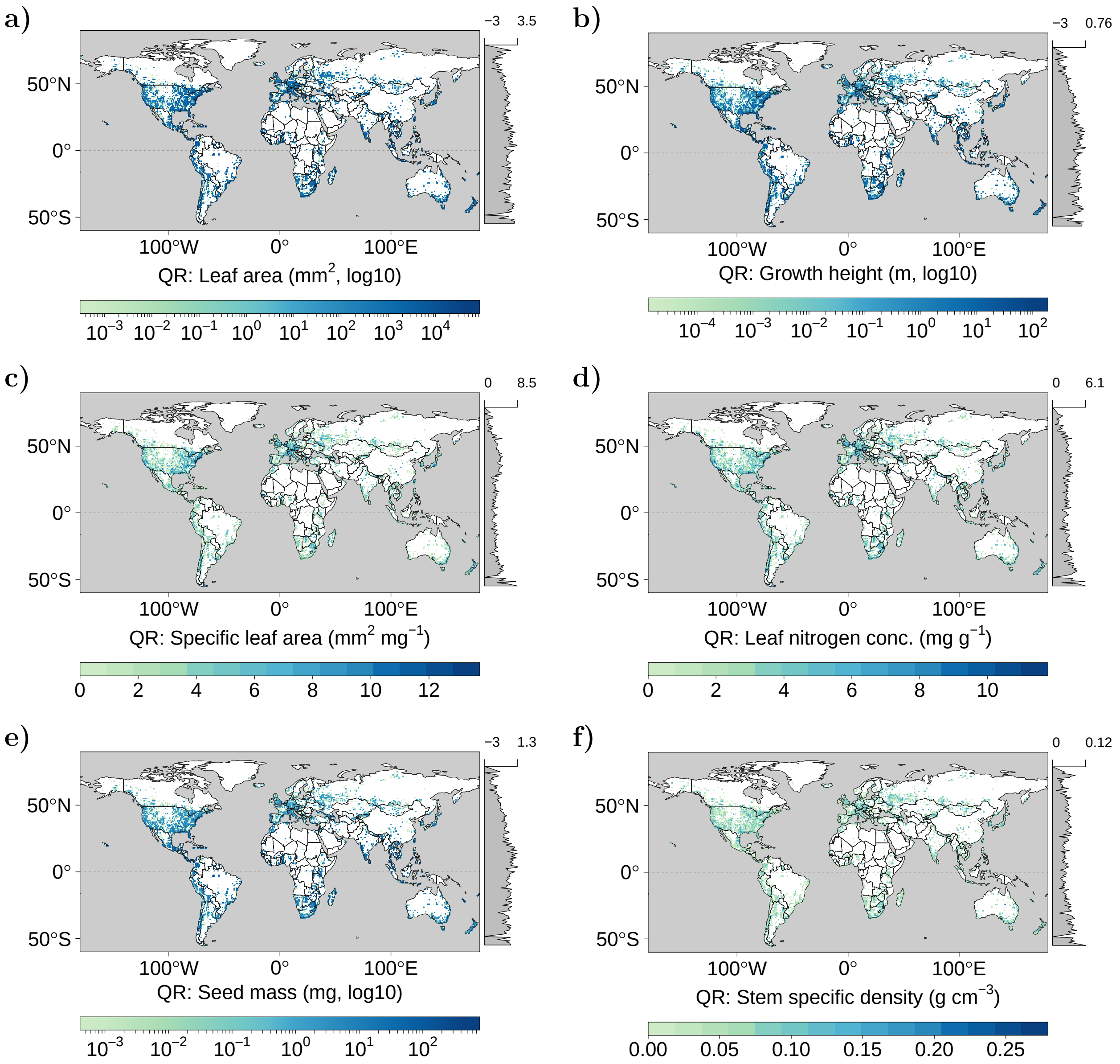

Supplementary Figure 8: Global trait quantile range maps. Global maps of quantile range (.9 quantile - .1 quantile) of plant functional traits on leaf area (**a**), growth height (**b**), specific leaf area (**c**), leaf nitrogen concentration (**d**), seed mass (**e**), stem specific density (**f**) including latitudinal distribution. Values of leaf area, growth height and seed mass were $\log_{10}$-transformed for improved visualisation.

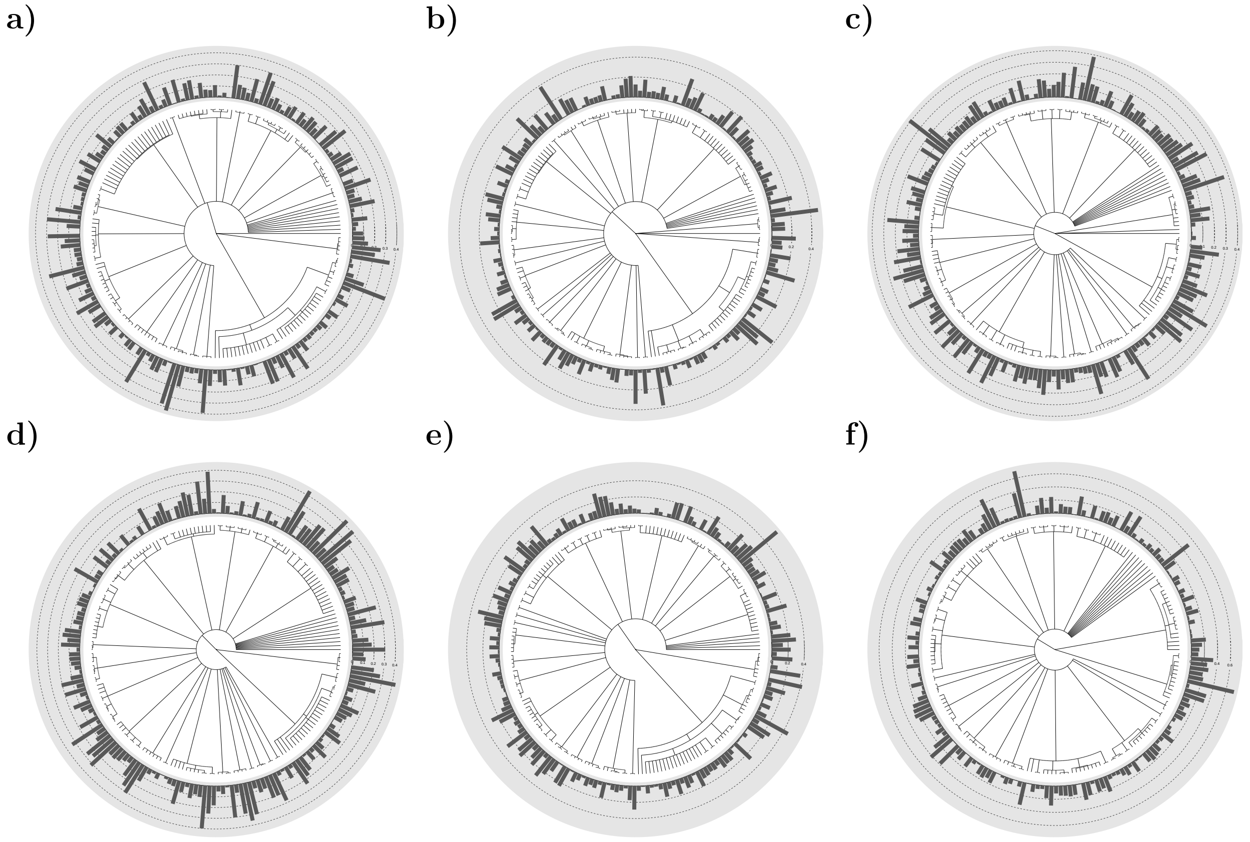

Supplementary Figure 9: Phylogenetic trees. a-f, Phylogenetic trees and model errors for the evaluation data (200 images per trait) of leaf area (**a**), growth height (**b**), specific leaf area (**c**), leaf nitrogen concentration (**d**), seed mass (**e**), stem specific density (**f**). Bars indicate mean absolute error (MAE) for each trait prediction.

Supplementary Table 1: Description of plant functional traits. Short description of each of the six plant functional traits incorporated in this study, including its unit as derived from the TRY database[5]. LA, leaf area, GH, growth height, SLA, specific leaf area, LNC, leaf nitrogen concentration, SM, seed mass, SSD, stem specific density.

Trait	Unit in TRY	Description	Ecological significance
LA	mm^2	Leaf area.<br>In case of compound leaves:<br>whole leaf, including petiole	Competitive ability
GH	m	Plant height.<br>Plant height in generative growth stage.	Competitive ability
SLA	$mm^2\ mg^{-1}$	Specific leaf area.<br>Leaf area per leaf dry mass.<br>Inverse of leaf mass per area (LMA)	Leaf economics
LNC	$mg\ g^{-1}$	Leaf nitrogen concentration per leaf dry mass.<br>Ratio of mass of nitrogen atoms in a leaf/leaflet<br>per respective unit dry mass	Leaf economics
SM	mg	Seed dry mass.<br>The mass of one seed assessed after drying.	Competitive ability
SSD	$g\ cm^{-3}$	Stem specific density.<br>Syn.: wood density.<br>Stem dry mass per stem fresh volume	Competitive ability

Supplementary Table 2: Numerical results of the model setups. Normalised mean absolute error (NMAE) in % as well as R^2 for each of the plant functional traits for each model setup. NMAE is the mean absolute error of the test dataset divided by the range of the target values of the test dataset, expressed as a percentage. The same data is visualised in Figure 2. LA, leaf area; GH, growth height; SLA, specific leaf area; LNC, leaf nitrogen concentration; SM, seed mass; SSD, stem specific density.

Step	LA		GH		SLA		LNC		SM		SSD	
	NMAE	R^2	NMAE	R^2	NMAE	R^2	NMAE	R^2	NMAE	R^2	NMAE	R^2
Baseline	11.83	.2932	11.6	.4699	12.07	.1726	13.19	.053	12.27	.1926	13.58	.0112
Plasticity	11.16	.3269	10.94	.5078	11.84	.1941	13.12	.06	12.34	.1817	13.58	.0121
Worldclim	10.08	.4467	10.63	.5401	11.26	.2769	12.92	.1084	12.03	.2304	12.31	.1709
Ensemble	9.58	.4906	10.21	.5728	11.33	.2849	12.44	.1567	11.74	.2569	12.19	.195

Supplementary Table 3: Mean trait prediction errors in original unit for training data of 3-fold cross-validation. Prediction errors in original unit for each of the plant functional traits for all of the training data of the 3-fold cross-validation. LA, leaf area; GH, growth height; SLA, specific leaf area; LNC, leaf nitrogen concentration; SM, seed mass; SSD, stem specific density.

	LA [mm^2]	GH [m]	SLA [mm^2/mg]	LNC [mg/g]	SM [mg]	SSD [g/cm^3]
Minimum	3.797	0.007	2.022	5.34	0.001	0.17
Maximum	226,971	80	92.77	68.983	7592.647	1.39
Mean error	3525.25	3.39	6.71	6.1	88.41	0.13

Supplementary Table 4: Significance test of evaluation dataset. Results of pairwise Student's t-tests (two-sided) with Bonferroni adjustment concerning image quality (low, medium, high), growth form (non-woody, woody) and image-target distance (< 1m, 1-5m, > 5m). Columns 'Group 1' and 'Group 2' indicate the two categories being compared, while n_1 and n_2 are the number of data points in the respective group, and p_{adj} is the Bonferroni-adjusted p-value of Student's t-test.

Group 1	Group 2	n_1	n_2	p_{adj}
Low	Medium	6	79	.52
Low	High	6	1,115	.28
Medium	High	79	1,115	1
Non-woody	Woody	549	651	.15
<1m	1-5m	762	347	.6
<1m	>5m	762	91	.86
1-5m	>5m	347	91	1

Supplementary Table 5: Results of test for phylogenetic autocorrelation. Number of samples for each trait (n), Pagel's λ test statistic and the corresponding p-value for the model errors of the evaluation datasets are shown for leaf area (LA), growth height (GH), specific leaf area (SLA), leaf nitrogen concentration (LNC), seed mass (SM) and stem specific density (SSD).

	LA	GH	SLA	LNC	SM	SSD
n	187	196	195	194	199	189
Pagel's λ	.03266	.00006	.00006	.04578	.11179	.17574
p-value	.567	1	1	.381	.132	.002

Supplementary Information 1 Phylogenetic Signal

We assessed the phylogenetic signal, i.e. the occurrence of phylogenetic autocorrelation, in the models' predictions using the 200 images of each trait contained in the evaluation dataset. We used the mean absolute error (i.e., the mean difference of predictions and targets for each species represented in the respective dataset) as dependent variable. The phylogenetic information contained in the iNaturalist database, including kingdom, phylum, order, class, family and genus for each species, was used as independent variable. We chose Pagel's λ as an indicator of phylogenetic signal, as it has shown to perform better than other indices and independent of species numbers[6]. Calculating Pagel's λ (Supplementary Table 5) and plotting the MAE distributions with phylogenetic trees (Supplementary Fig. 9) was done using R package 'phylosignal' (version 1.3)[7]. Results show that with the exception of SSD, neither of the plant traits predicted in this study shows a significant phylogenetic autocorrelation with $p < .05$ (Supplementary Table 5).

Supplementary Information 2 Evaluation Criteria

In this section, we describe the criteria for the visual interpretation regarding the evaluation process of our model results. The image quality and image-target distance were interpreted visually, whereas the 'woody vs. non-woody' category information was derived from the plant growth form data in the TRY database.

The image quality was assessed by three different categories. Images that were almost completely blurred in such a way that the target species (including all of its organs) appeared hardly recognisable in most of the image were categorized as 'low' quality images (Supplementary Fig. 7 a). The 'medium' quality was allocated to images containing target species that were slightly blurred in some parts of the image, with organs that could clearly be distinguished (Supplementary Fig. 7 b). Images with 'high' quality contained target species that were focused, and all organs that appeared in the image were clearly recognisable (Supplementary Fig. 7 c-f). The background was permitted to be unfocused to qualify for category 'high' (Supplementary Fig. 7 e-f).

Regarding image-target distance, the chosen categories were 'less than 1 m', 'between 1 and 5 m' and 'more than 5 m'. The first category contained images with close-up photographs, e.g. focusing specifically on a plant's organ such as its flower (Supplementary Fig. 7 e). The second category included target species located in a medium distance to the photographer, but in such a way that full-grown trees could not be captured completely. Herbs and shrubs, however, usually appeared almost completely on these photographs (Supplementary Fig. 7 b-c). The third category comprising images with target species furthest away (>5 m) contained photographs showing a significant proportion even of full-grown trees (Supplementary Fig. 7 d).

References

1. Boonman, C. C. F. *et al.* Assessing the reliability of predicted plant trait distributions at the global scale. *Glob. Ecol. Biogeogr.* **29,** 1034–1051 (2020).

2. van Bodegom, P. M., Douma, J. C. & Verheijen, L. M. A fully traits-based approach to modeling global vegetation distribution. *Proc. Natl. Acad. Sci. USA* **111,** 13733–13738 (2014).

3. Butler, E. E. *et al.* Mapping local and global variability in plant trait distributions. *Proc. Natl. Acad. Sci. USA* **114,** E10937–E10946 (2017).

4. Moreno-Martínez, Á. *et al.* A methodology to derive global maps of leaf traits using remote sensing and climate data. *Remote Sens. Environ.* **218,** 69–88 (2018).

5. Kattge, J. *et al.* TRY plant trait database - enhanced coverage and open access. *Glob. Chang. Biol.* **26,** 119–188 (2020).

6. Münkemüller, T. *et al.* How to measure and test phylogenetic signal. *Methods Ecol. Evol.* **3,** 743–756 (2012).

7. Keck, F., Rimet, F., Bouchez, A. & Franc, A. phylosignal: an R package to measure, test, and explore the phylogenetic signal. *Ecol. Evol.* **6,** 2774–2780 (2016).
